# Liraglutide restores chronic ER stress, autophagy impairments and apoptotic signalling in SH-SY5Y cells

**DOI:** 10.1038/s41598-017-16488-x

**Published:** 2017-11-23

**Authors:** Theodora Panagaki, Maria Michael, Christian Hölscher

**Affiliations:** Biomedical & Life Sciences Division, Lancaster University, Lancaster, LA1 4YG UK

## Abstract

Growing evidence suggests that agonists of glucagon-like peptide (GLP-1) receptor exert neuroprotective and neurorestorative effects across a range of experimental models of neuronal degeneration, and, recently, a pilot clinical trial of Liraglutide in Alzheimer’s disease patients showed improvements in cerebral glucose consumption that signifies disease progression. However, the exact underlying mechanism of action remains unclear. Chronic endoplasmic reticulum (ER) stress has recently emerged as a mechanism for neuronal injury, rendering it a potent therapeutic target for acute and chronic neurodegenerative disorders. Here, we investigate the neuroprotective effects of Liraglutide along with the signalling network against prolong ER stress and autophagy impairments induced by the non-competitive inhibitor of sarco/ER Ca^2+^-ATPase, thapsigargin. We show that Liraglutide modulates the ER stress response and elicits ER proteostasis and autophagy machinery homeostasis in human SH-SY5Y neuroblastoma cell line. These effects correlate with resolution of hyper-activity of the antioxidant Nrf2 factor and restoration of the impaired cell viability and proliferation. Mechanistically, Liraglutide engages Akt and signal transducer and activator of transcription 3 (STAT3) signalling to favour adaptive responses and shift cell fate from apoptosis to survival under chronic stress conditions in SH-SY5Y cells.

## Introduction

Neuronal injury owing to chronic stress of the endoplasmic reticulum (ER) is increasingly being recognised as a common contributor to amyotrophic lateral sclerosis (ALS), Alzheimer’s disease (AD), Parkinson’s disease (PD), ischaemic stroke and traumatic brain injury (TBI)^1–5^. Shared among these seemingly dissimilar neurological disorders is the presence of intracellular and/or extracellular conditions that perturb signalling and handling of calcium, protein folding processes and autophagic machinery, generating a vicious circle of irremediable ER stress^1–5^. The ER is a multifunctional signalling organelle that orchestrates calcium homeostasis and metabolic processes, including gluconeogenesis and the biosynthesis of autophagosomes in the cell. It additionally is the fundamental intracellular compartment for the synthesis, maturation, quality control and delivery of the secretory and membrane proteins^6^. Much physiological and pathological stimuli can alter the protein folding at the ER, triggering a rise in the unfolded or misfolded protein load in the organelle lumen, a cellular state referred to as ER stress^7^. In turn, the cell activates an adaptive signalling network, known as the unfolded protein response (UPR). The UPR essentially engages the three ER-resident transmembrane stress transducers – protein kinase RNA-like ER kinase (PERK), activating transcription factor 6 (ATF6) and inositol-requiring enzyme 1 (IRE1) – to safeguard proteostasis through attenuation of global protein synthesis and transcriptional induction of genes functioning as ER chaperones, and degrade the abnormal through the proteasome (ER-associated degradation) and lysosome-mediated autophagy^2,6,8^. However, under persistent and unsurmountable ER stress, the UPR adapts its dynamics and drives cells towards suicide through diverse but often overlapping mechanisms, including the induction of proteases, kinases, the transcription of CAAT/enhancer-binding protein (C/EBP) homologous protein (CHOP) and Bcl-2 family members along with their mediators^6,8^. It is therefore intuitive that therapeutic interventions which resolve UPR and promote a balance between protein generation and degradation crucial for proteostasis may significantly benefit the clinical outcome of acute and chronic neurodegenerative disorders^1,3–5^. In this regard, we have focused our research efforts on investigating the restorative effects of the neuroprotective glucagon-like peptide 1 (GLP-1) analogue Liraglutide against chronic ER stress and autophagy dysfunction in SH-SY5Y neuroblastoma cell line.

The incretin hormone GLP-1 is best known for regulating glucose homeostasis and insulin signalling and biosynthesis in response to food ingestion. As such, GLP-1 mimetics are currently approved for the treatment of type 2 diabetes mellitus (T2DM). Apart from their glucose-dependent pancreatic effects, GLP-1 mimetics cross the blood brain barrier and modulate multiple cellular processes within the central nervous system (CNS), including synaptogenesis, neuronal energetics, memory formation and inflammatory responses^9,10^. For instance, intraperitoneal administration of Liraglutide has rescued cognitive and synaptic plasticity deficits, halted excessive synaptic loss, enhanced mitochondria biogenesis and clearance of aggregated proteins and/or mitigated microglia activation and inflammation in a transgenic APP/PS1 mouse model of AD^11,12^, in a 1-methyl-4-phenyl-1,2,3,6-tetrahydropyridine (MPTP) model of PD^13^, in a transgenic mouse model of dementia-related tauopathy^14^, in a rat model of middle cerebral artery occlusion^15,16^, and in a mouse model of mild TBI^17^. In line with the *in-vivo* data, Liraglutide and other GLP-1 mimetics have protected cultured neurons and neuronal cell lines from hypoxia, oxidative stress and excitotoxic injury^17–21^. Notably, in a pilot clinical trial, Liraglutide has rescued the decline of cerebral glucose consumption in AD patients, which signifies energy metabolism in brain areas that have been correlated with cognitive decline in AD and therefore disease progression^22^. Additionally, a pilot open-label clinical trial of the GLP-1 analogue Exenatide has demonstrated persistent improvements in cognitive and motor function of Parkinson’s patients^23,24^. A recently-published phase II placebo-controlled double-blind trial has similarly shown that Exenatide halts PD progression and thus confirmed the aforementioned preliminary data^25^. The neuroprotective effects of GLP-1 mimetics lie downstream of the induction of the GLP-1 receptors (GLP-1Rs)^9,10^. Indeed, GLP-1R overexpression in hippocampus augments spatial learning and memory performance *in vivo*, effects that are blocked in the presence of a GLP-1R antagonist^26^. Conversely, GLP-1R-deficient mice feature impaired hippocampal synaptic plasticity^27^ accompanied by decrements in associative contextual^26^ and spatial learning^27^, and increased susceptibility to kainic acid-induced seizures and neuronal degeneration in the hippocampus^26^, as compared to wild-type controls. GLP-1Rs are widely expressed in the brain and mediate survival and trophic signals via the G*α*-protein, culminating into the stimulation of adenyl cyclase, Akt and mitogen-activated protein kinase (MAPK) pathways^9,10^. However, whether GLP-1R agonists regulate highly conserved cellular mechanisms, such as the UPR and protein quality control machinery, and engage additional signalling pathways to the aforementioned to promote neuronal survival and tissue repair remains largely unexplored.

Here we show that chronic Liraglutide treatment modulates UPR signalling, restores ER proteostasis, and promotes autophagic machinery homeostasis to shift cell fate from apoptosis to survival in the SH-SY5Y human neuroblastoma cells. To gain an integrated view of the Liraglutide-induced neuroprotection following aberrant ER stress and autophagy response, we correlate these effects to the antioxidant defensive Nrf2 factor activity and the intracellular signalling ‘pool’. In line with previous findings, we report that the GLP-1R stimulation induces Akt to favour adaptive responses following cellular stress. Our study further unravels that the signal transducer and activator of transcription 3 (STAT3) mediates survival signals downstream of GLP-1R stimulation upon irremediable neuronal ER stress. Taken together, our findings provide additional evidence for the beneficial effects of GLP-1R signalling in neurodegenerative disorders and deepen our understanding of the underlying mechanism.

## Results

### Liraglutide rescues thapsigargin-induced cytotoxicity and cell-growth arrest

**Figure 1:**
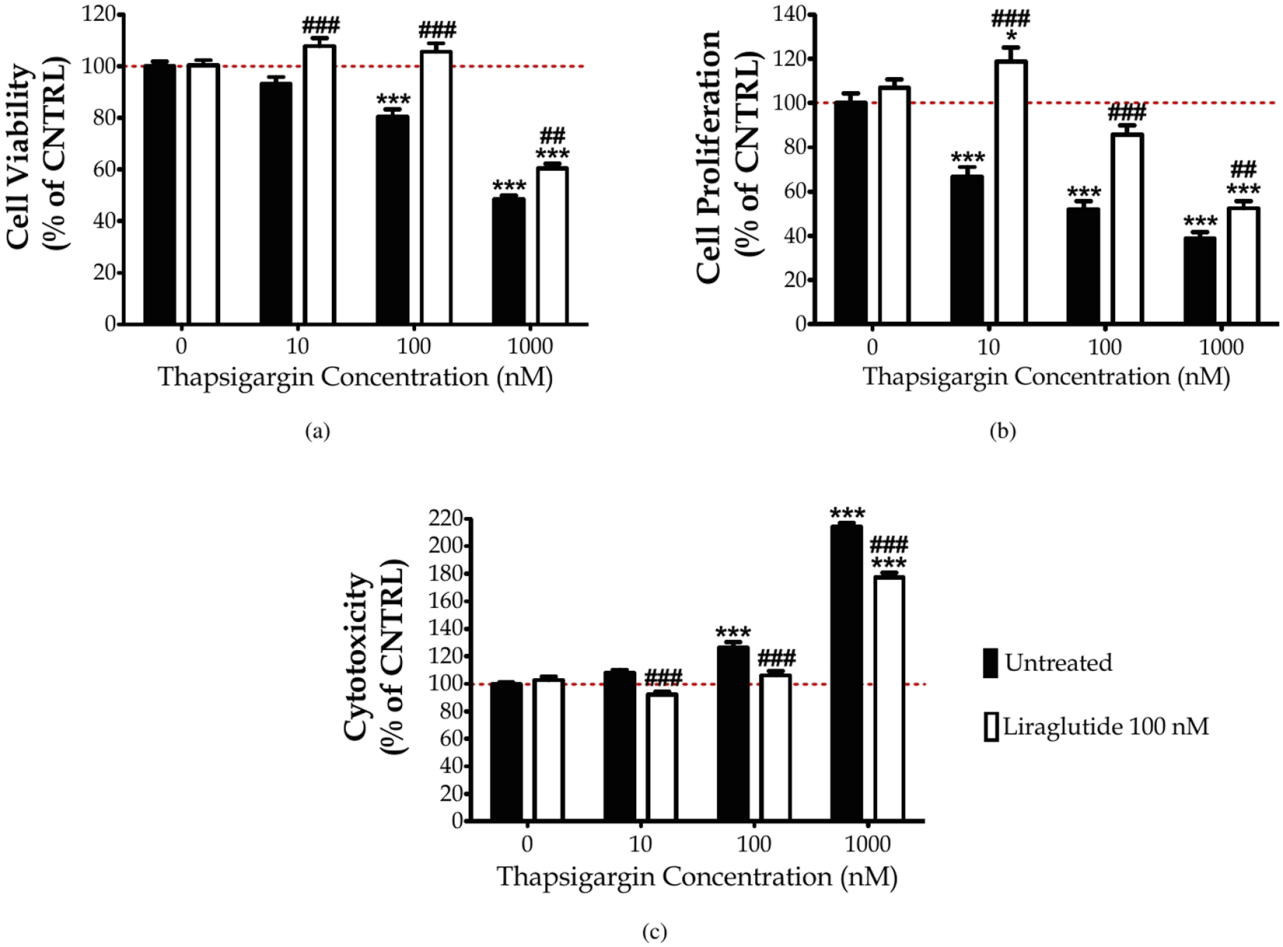
Liraglutide restores abnormal cytotoxicity and ameliorates impaired viability and proliferation in the neuroblastoma SH-SY5Y cell line upon persistent ER stress. Twenty-four hours post seeding, SH-SY5Y cells serum starved for 8 h and treated with 0, 10, 100 and 1000 nM of thapsigargin for 16 h, in the presence or absence of 100 nM Liraglutide. Cells were then assayed for XTT metabolisation [(**a**)] and BrdU incorporation [(**b**)] to assess cell viability and proliferation, respectively. Cell supernatant was collected and processed for LDH activity [(**c**)] to determine cytotoxicity. Each bar represents mean ± SEM from four independent experiments. All cell treatments were performed in sextuplicate per plate per experiment. Data is expressed as a percentage of the control (CNTRL; unstressed/untreated conditions). Data was analysed by one- and two-way ANOVA, followed by *post hoc* Bonferroni’s multiple comparison t-test (^*^ *p* ≤ 0.05 & ^***^ *p* ≤ 0.001 compared to CNTRL; ^##^ *p* ≤ 0.01 & ^###^ *p* ≤ 0.001 compared to the corresponding thapsigargin-treated cells).

First, we elucidated the cytoprotective effect of Liraglutide in the SH-SY5Y human neuroblastoma cells from chronic ER stress by thapsigargin. Thapsigargin is a naturally occurring sesquiterpene lactone that selectively inhibits sarcoplasmic/ER Ca^2+^–ATPase (SERCA), triggering a transient increase in the cytosolic calcium and depleting ER calcium stores^28^. Cells were treated with 0, 10, 100 and 1000 nM of thapsigargin in the presence or absence of 100 nM Liraglutide for 16 h, and processed for XTT, BrdU and LDH assays to assess cell viability, proliferation and cytotoxicity, respectively. One-way ANOVA analysis reveals overall significant differences in cell viability (*F*_(7,442)_ = 79.59, *p* ≤ 0.001), proliferation (*F*_(7,355)_ = 48.98, *p* ≤ 0.001) and cytotoxicity (*F*_(7,436)_ = 255.2, *p* ≤ 0.001). *Post-hoc* analysis with Bonferroni correction further reveals that thapsigargin impairs cell viability in a dose-dependent fashion; it triggers an approximately 7%, 20% (*p* ≤ 0.001) and 50% (*p* ≤ 0.001) decline in the number of viable, metabolically active cells that were accordingly challenged with 10, 100 and 1000 nM of the stressor, with respect to control conditions [**Subigure 1(a)**]. A dose-dependent cell growth arrest and membrane perturbation accompanies the suppressed cell viability. Specifically, the percentage of proliferating cells approximately decreases by 30% (*p* ≤ 0.001), 50% (*p* ≤ 0.001) and 60% (*p* ≤ 0.001) in microcultures exposed to 10, 100 and 1000 nM of thapsigargin, respectively, when compared to control conditions [Fig. 1(b)]. Conversely, LDH release progressively increases by 8%, 30% (*p* ≤ 0.001) and 120% (*p* ≤ 0.001) in the supernatant of microcultures received the aforementioned stressor doses [Fig. 1(c)]. Two-way ANOVA analysis demonstrates that Liraglutide co-treatment significantly rescues abnormal LDH activity (*F*_(3,436)_ = 16.91, *p* ≤ 0.001) and ameliorates cell viability (*F*_(3,442)_ = 8.14, *p* ≤ 0.001) and proliferation (*F*_(3,355)_ = 11.75, *p* ≤ 0.001) impairments following chronic SERCA inhibition [Fig. 1]. Interestingly, Liraglutide approximately induces a 25% and 35% rise in the number of metabolically active and proliferating cells when stressed with 100 nM of thapsigargin, and renders them indifferent from the control conditions [Fig. 1(a), (b)]. Similarly, Liraglutide co-treatment normalises the abnormal LDH activity in the SH-SY5Y microcultures received 100 nM of thapsigargin [Fig. 1(c)]. Therefore, we consider 100 nM of thapsigargin stress for further studies where Liraglutide fully restores cell physiology.

### Liraglutide resolves UPR and brings homeostasis in the protein folding machinery of ER

**Figure 2:**
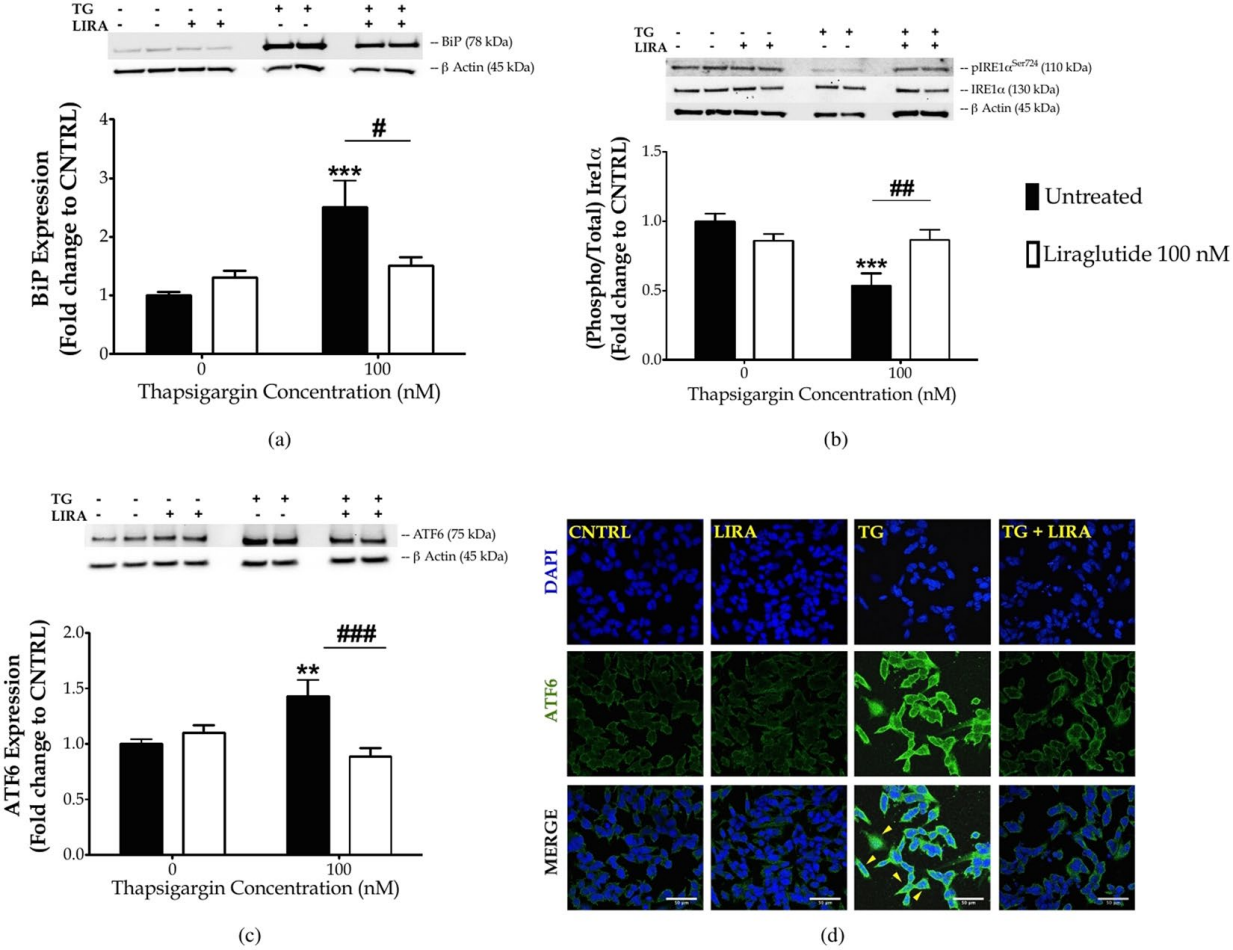
Liraglutide resolves UPR in the neuroblastoma SH-SY5Y cell line. Twenty-four hours post seeding, SH-SY5Y cells were serum starved for 8 h and treated with 0 or 100 nM of thapsigargin (TG) for 16 h, in the presence or absence of 100 nM Liraglutide (LIRA). Cells were harvested, and the expression of BiP [**(a)**] and ATF6 [**(c)**], as well the protein levels of total and phosphorylated IRE1*α* [**(b)**] were determined by western blotting. *β*-Actin was used as the loading control to all western blot analyses. Each bar represents mean ± SEM from four independent experiments. Data is expressed as fold change to the control (CNTRL; unstressed/untreated conditions). Data was analysed by one- and two-way ANOVA, followed by *post hoc* Bonferroni’s multiple comparison t-test (^**^ *p* ≤ 0.01 & ^***^ *p* ≤ 0.001 compared to CNTRL; ^#^ *p* ≤ 0.05, ^##^ *p* ≤ 0.01 & ^###^ *p* ≤ 0.001 compared to the corresponding thapsigargin-treated cells). [**(d)**] Representative pictures of SHSY5Y cells immunolabelled for ATF6 post thapsigargin and liraglutide treatments. Scale bars: 50 μm.

**Figure 3:**
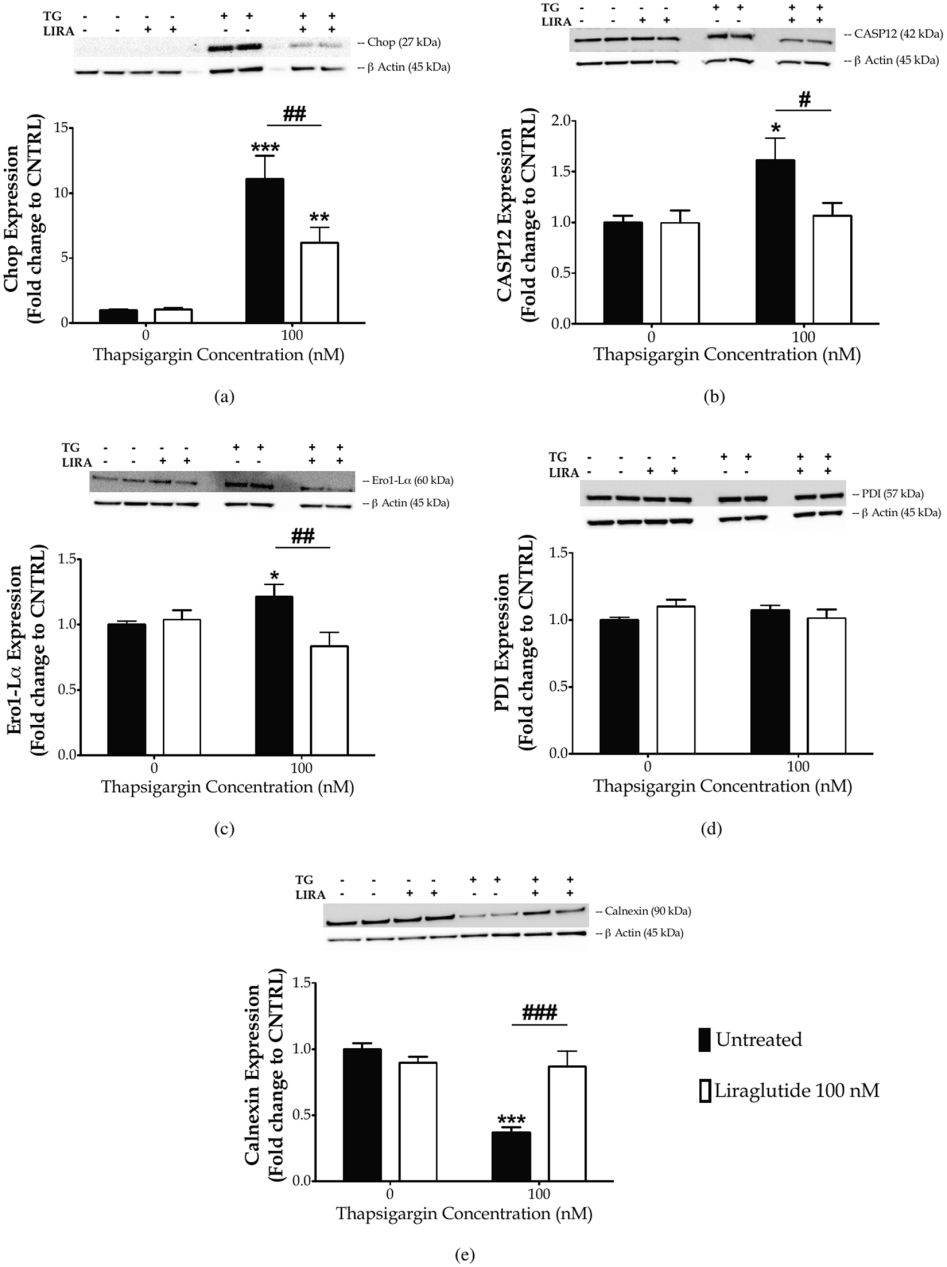
Liraglutide ameliorates the ectopic expression of pro-apoptotic UPR mediators and promotes ER proteostasis in the neuroblastoma SH-SY5Y cell line upon persistent ER stress. Twenty-four hours post seeding, SH-SY5Y cells were serum starved for 8 h and treated with 0 or 100 nM of thapsigargin for 16 h, in the presence or absence of 100 nM Liraglutide. Cells were harvested, and the expression of CHOP [(**a**)], caspase 12 (CASP12) [(**b**)], ER oxidoreductase 1 alpha (Ero1-L*α*) [(**c**)], protein disulfide isomerase [PDI; (**d**)] and of calnexin [(**e**)] were determined by western blotting. *β*-Actin was used as the loading control to all western blot analyses. Each bar represents mean ± SEM from four independent experiments. Data is expressed as fold change to the control (CNTRL; unstressed/untreated conditions). Data was analysed by one- and two-way ANOVA, followed by *post hoc* Bonferroni’s multiple comparison t-test (^*^ *p* ≤ 0.05, ^**^ *p* ≤ 0.01 & ^***^ *p* ≤ 0.001 compared to CNTRL; ^#^ *p* ≤ 0.05, ^##^ *p* ≤ 0.01 & ^###^ *p* ≤ 0.001 compared to the corresponding thapsigargin-treated cells).

Next, we investigated whether Liraglutide could regulate the UPR to promote neuroprotection in SH-SY5Y cells. Cells were treated with 0 and 100 nM of thapsigargin in the presence or absence of 100 nM Liraglutide for 16 h, and processed for immunoblotting analysis of hallmark ER stress-related signalling molecules. One-way ANOVA analysis demonstrates overall significant differences in the binding immunoglobulin protein (BiP) (*F*_(3,28)_ = 27.90, *p* ≤ 0.001), full-length ATF6 (*F*_(3,28)_ = 6.267, *p* ≤ 0.01), IRE1*α* activation (*F*_(3,27)_ = 8.683, *p* ≤ 0.001), CHOP (*F*_(3,28)_ = 20.66, *p* ≤ 0.001), and active caspase 12 (CASP12) (*F*_(3,28)_ = 4.185, *p* ≤ 0.05). In particular, chronic thapsigargin treatment triggers a 1.5-fold increase in BiP expression [Fig. 2(a)] that indicates ER stress^29^, and a significant increase in the full-length ATF6 levels (*p* ≤ 0.01), as shown in Fig. 2(c). Upon ER stress, ATF6 translocates to Golgi; there, it undergoes proteolytic cleavage at a juxtamembrane site to generate an active cytosolic ATF6 fragment that subsequently migrates into the nucleus^7^. Although we could not consistently detect the active, 50- ATF6 proteolytic fragment among the four immunoblotting experiments analysed (data not shown), confocal imaging has revealed a nuclear accumulation of this factor following chronic thapsigargin treatment in SH-SY5Y cells [Fig. 2(d)] that signifies ATF6 activation. Furthermore, chronic thapsigargin treatment significantly precludes the activating phosphorylation of IRE1*α* at the serine 724 residue (Ser724) (*p* ≤ 0.001), as compared to control conditions [Fig. 2(b)]. It additionally potentiates a 10-fold increase in CHOP (*p* ≤ 0.001) and almost doubles the active CASP12 fragment at 42 kDa (*p* ≤ 0.05), as shown in Fig. 3(a)–(b). Intriguingly, Liraglutide normalises abnormal BiP [Fig. 2(a)] and ATF6 [Fig. 2(c)] protein expression, and rescues IRE1*α* [Fig. 2(b)] and ATF6 activation [Fig. 2(d)]. Moreover, Liraglutide restores CASP12 up-regulation [Fig. 3(b)] and significantly ameliorates the ectopic CHOP expression by halving protein levels of this transcription factor (*p* ≤ 0.01), though they remained significantly elevated when compared to control conditions (*p* ≤ 0.01) [Fig. 3(a)].

To correlate the restorative effects of Liraglutide on the UPR with the ER proteostasis, we evaluated the protein expression of major protein quality-control chaperones, *i.e*., the ER oxidoreductase 1*α* (Ero1-L*α*) [Fig. 3(c)], protein disulphide isomerase (PDI) [Fig. 3(d)] and calnexin [Fig. 3(e)]. One-way ANOVA analysis reveals overall significant differences in Ero1-L*α* (*F*_(3,28)_ = 3.668, *p* ≤ 0.05) and calnexin (*F*_(3,28)_ = 11.62, *p* ≤ 0.001) but not in PDI expression (*F*_(3,28)_ = 1.053, *p* = 0.3846). *Post-hoc* analysis shows that chronic thapsigargin treatment significantly up-regulates Ero1-L*α* protein levels (*p* ≤ 0.05) whilst down-regulates calnexin by approximately 60% (*p* ≤ 0.001). Notably, Liraglutide co-treatment normalises abnormal protein expression of Ero1-L*α* and calnexin, as shown in Fig. 3(c) and (e), respectively.

### Liraglutide rescues thapsigargin-induced autophagy impairments

**Figure 4:**
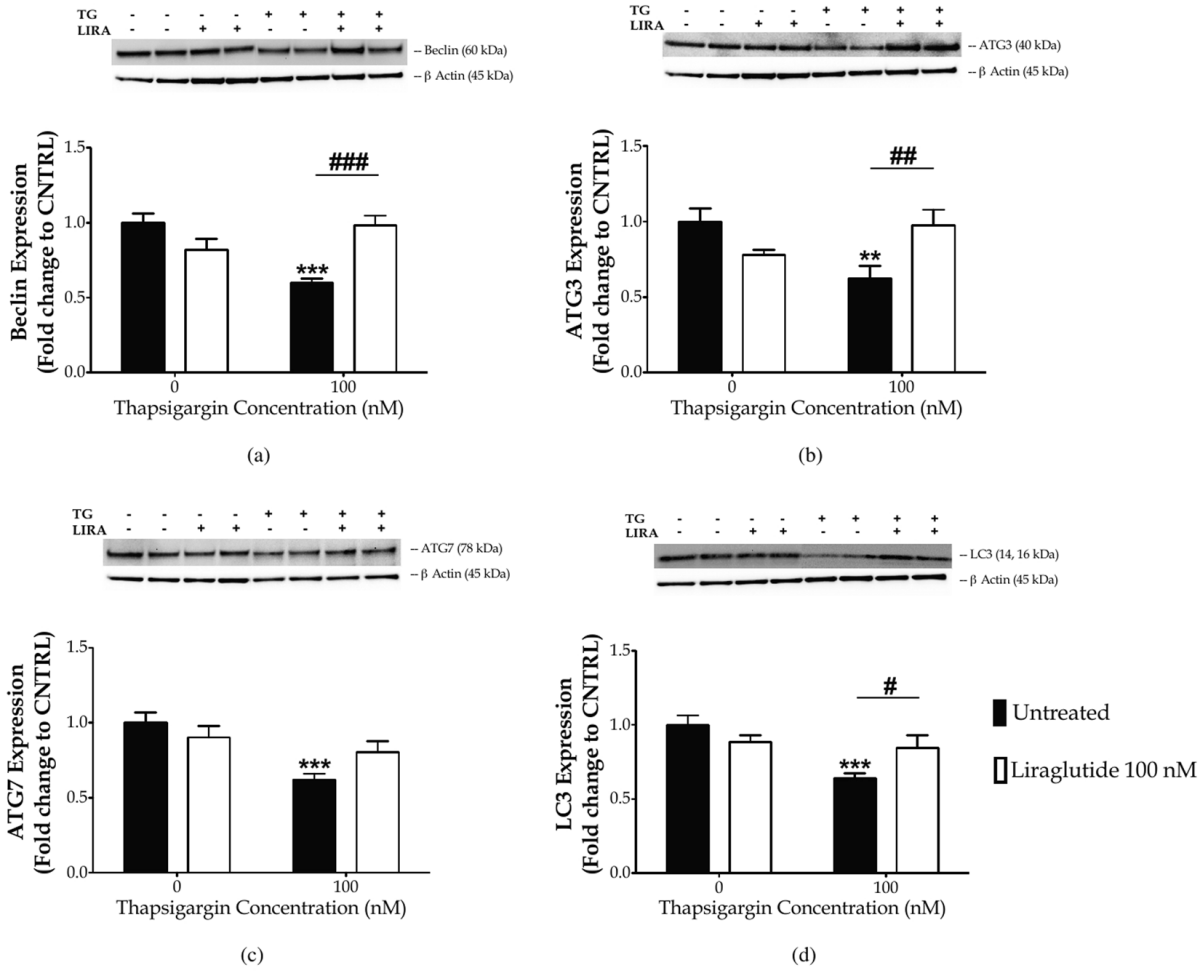
Chronic disturbance of ER calcium homeostasis diminishes “core” Atg proteins required for autophagosome formation. Liraglutide rescues autophagy impairments in the neuroblastoma SH-SY5Y cell line upon persistent ER stress. Twenty-four hours post seeding, SH-SY5Y cells were serum starved for 8 h and treated with 0 or 100 nM of thapsigargin for 16 h, in the presence or absence of 100 nM Liraglutide. Cells were harvested, and the expression of beclin [(**a**)], ATG3 [(**b**)], ATG7 [(**c**)] and of LC3 [(**d**)] were determined by western blotting. *β*-Actin was used as the loading control to all western blot analyses. Each bar represents mean ± SEM from four independent experiments. Data is expressed as fold change to the control (CNTRL; unstressed/untreated conditions). Data was analysed by one- and two-way ANOVA, followed by *post hoc* Bonferroni’s multiple comparison t-test (^**^ *p* ≤ 0.01 & ^***^ *p* ≤ 0.001 compared to CNTRL; ^#^ *p* ≤ 0.05, ^##^ *p* ≤ 0.01 & ^###^ *p* ≤ 0.001 compared to the corresponding thapsigargin-treated cells).

ER-localised blocking of calcium flux has been previously shown to perturb autophagosome biogenesis^30^ and fusion with lysosome^31^, culminating into autophagy arrest^31,30^ that can, in turn, elicit or enhance ER stress^32^. In this light, we investigated the expression of a set of autophagy-related (Atg) proteins following chronic thapsigargin and Liraglutide treatment [Fig. 4]. Protein expression of beclin (*F*_(3,28)_ = 10.02, *p* ≤ 0.001), Atg3 (*F*_(3,28)_ = 4.78, *p* ≤ 0.01), Atg7 (*F*_(3,28)_ = 5.977, *p* ≤ 0.01) and of LC3 (*F*_(3,28)_ = 6.557, *p* ≤ 0.01) significantly differs among groups, as evident by one-way ANOVA analysis. *Post-hoc* analysis demonstrates that chronic thapsigargin treatment significantly suppresses beclin (*p* ≤ 0.001) [Fig. 4(a)], Atg3 (*p* ≤ 0.01) [Fig. 4(b)], Atg7 (*p* ≤ 0.001) [Fig. 4(c)], and LC3 (*p* ≤ 0.001) [Fig. 4(d)] protein expression. Two-way ANOVA analysis indicates that Liraglutide co-treatment significantly alleviates impairments in beclin (*F*_(1,28)_ = 23.07, *p* ≤ 0.001), Atg3 (*F*_(1,28)_ = 12.52, *p* ≤ 0.01) and LC3 (*F*_(1,28)_ = 7.43, *p* ≤ 0.05). Additionally, there is a trend of improvement by Liraglutide on the decreased Atg7 expression following thapsigargin treatment (*F*_(1,28)_ = 4.59, *p* ≤ 0.05), though it does not reach statistical significance when compared to the corresponding stress conditions in *post-hoc* analysis [Fig. 4(c)].

### Liraglutide normalises aberrant nuclear accumulation of Nrf2 following ER stress and autophagy impairments

**Figure 5:**
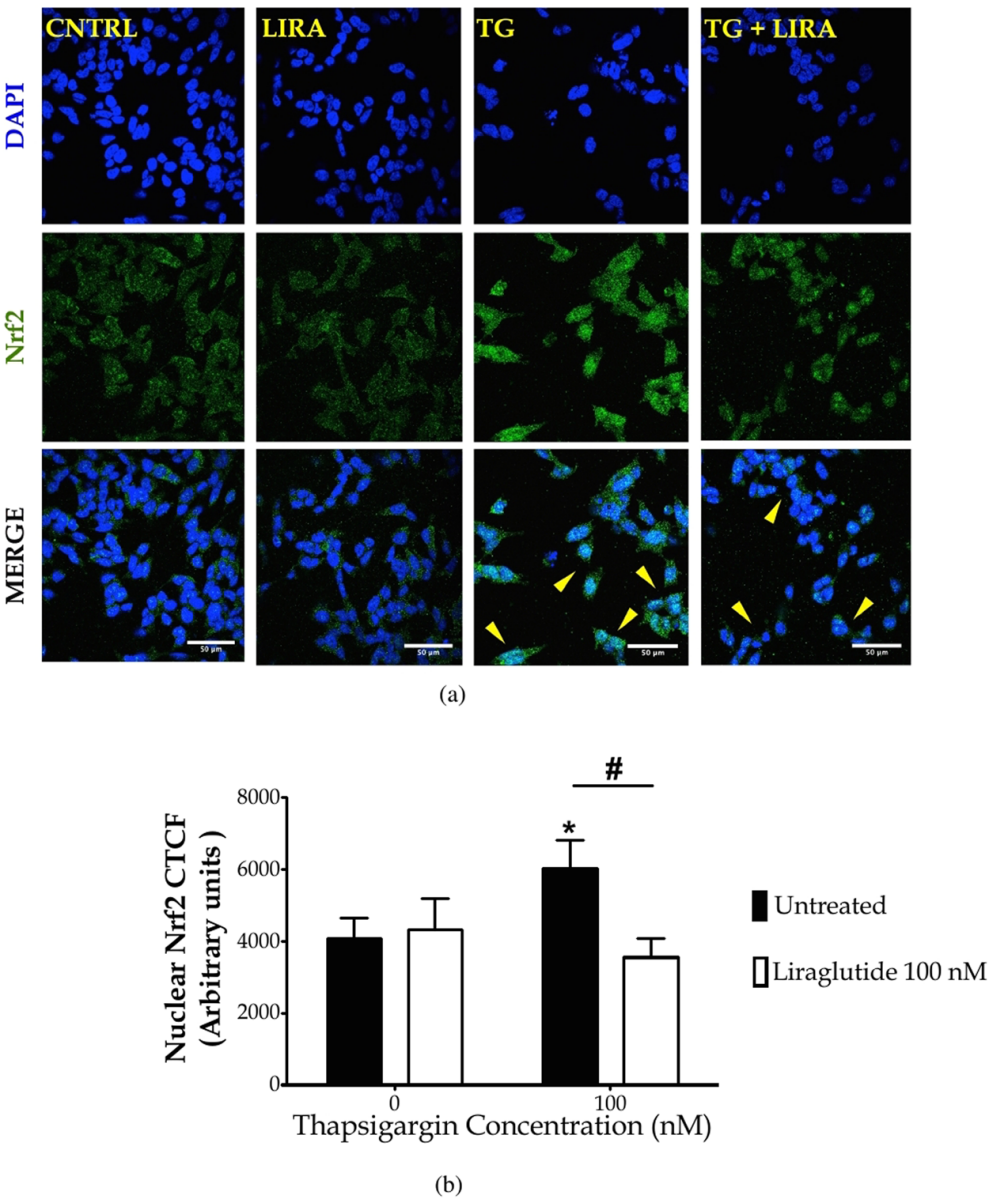
Autopphagy impairments along with UPR deregulation up-regulate the nuclear content of the nuclear factor erythroid 2-related factor 2 (Nrf2). Liraglutide restores Nrf2 signalling in the neuroblastoma SH-SY5Y cell line upon irremediable ER stress. Twenty-four hours post seeding, SH-SY5Y cells were serum starved for 8 h and treated with 0 or 100 nM of thapsigargin (TG) for 16 h, in the presence or absence of 100 nM Liraglutide (LIRA). Cells were paraformaldehyde-fixed, immunolabelled for Nrf2 and processed for confocal imaging at 40X magnification, as shown in the representative images [(**a**)]. Eight pictures were captured per experimental group per experiment for quantification. Image J was used to quantify corrected total cell fluorescence (CTCF) of the nuclear Nrf2 staining [(**b**)]. Each bar represents mean ± SEM from three independent experiments. Data was analysed by one- and two-way ANOVA, followed by *post hoc* Bonferroni’s multiple comparison t-test (^*^ *p* ≤ 0.05 compared to CNTRL; ^#^ *p* ≤ 0.05 compared to the corresponding thapsigargin-treated cells). Scale bars: 50 μm.

To further link aberrant UPR and autophagic machinery dysfunction to the antioxidant response in SH-SY5Y, we performed immunocytochemical analysis of the nuclear levels of the transcription factor Nrf2. Under basal conditions, Nrf2 is mainly localised to cytoplasm in a complex with the Kelch-like ECH-associated protein 1 (Keap1), an ubiquitin ligase substrate adaptor that targets Nrf2 for degradation^33^. Indeed, unstressed SH-SY5Y cells display a faint Nrf2-positive immunostaining that mainly resides in the peri-nuclear area [Fig. 5(a)]. However, in response to cellular stress, Nf2 dissociates from Keap1 and rapidly translocates into nucleus to confer its transcriptional activity^33^. Accordingly, we show that chronic thapsigargin treatment induces an aberrant nuclear accumulation of Nrf2 (*post hoc*; *p* ≤ 0.05) that is normalised by Liraglutide co-treatment [Fig. 5].

### Liraglutide restores impaired STAT3 activity and activates Akt signalling to ameliorate thapsigargin-induced apoptosis

**Figure 6:**
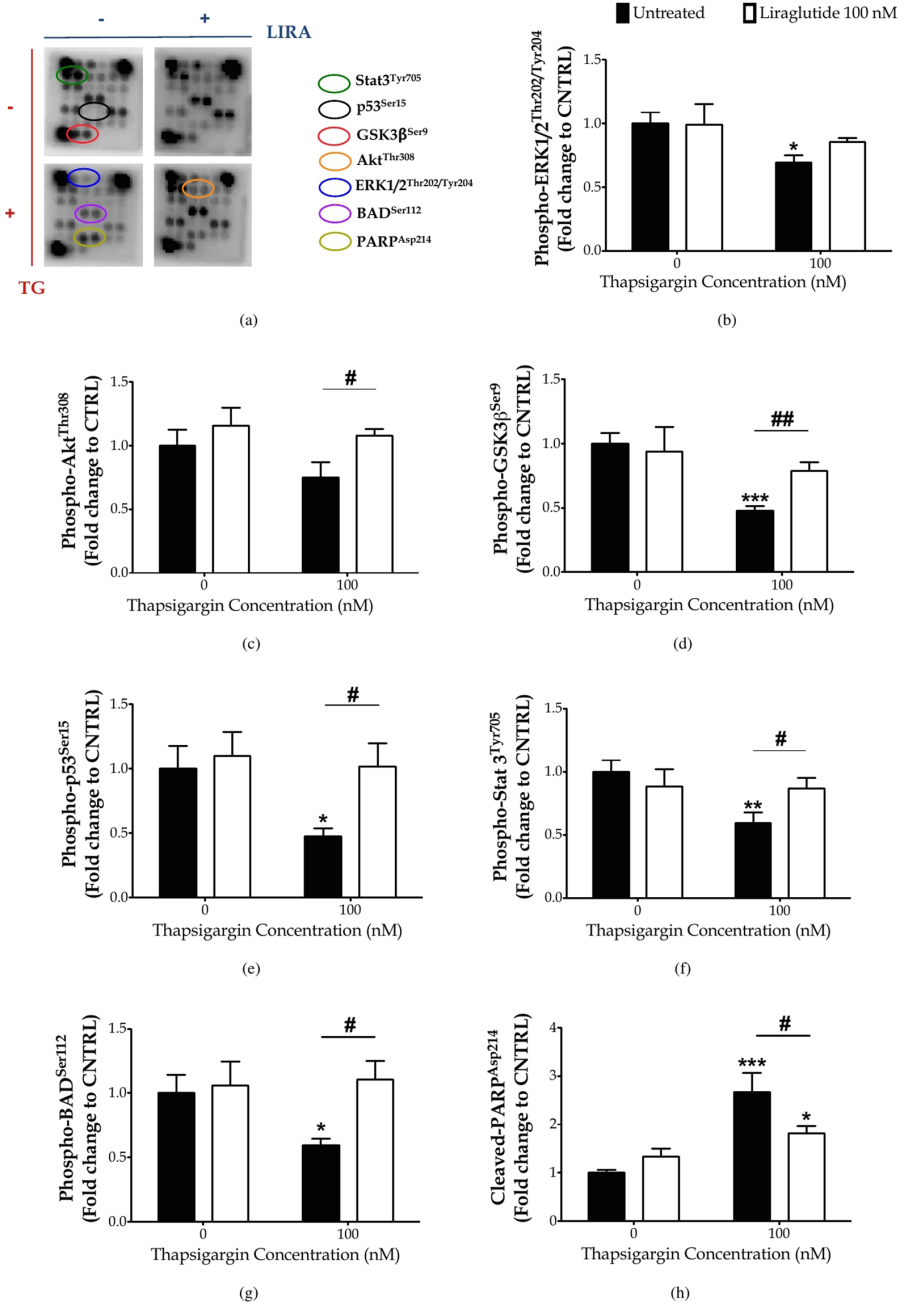
Chronic disturbance of ER calcium homeostasis induces PARP cleavage, diminishes inhibitory phosphorylation of the pro-apoptotic BAD protein and of the multifunctional GSK3b kinase, and impairs ERK and STAT3 signalling to promote genotoxicity and apoptosis. Liraglutide rescues PARP cleavage and activation of STAT3 and p53 kinases. It additionally induces Akt phosphorylation that relieves BAD and GSK3b activity to promote cell survival in the neuroblastoma SH-SY5Y cell line upon persistent ER stress. Twenty-four hours post seeding, SH-SY5Y cells were serum starved for 8 h and treated with 0 or 100 nM of thapsigargin for 16 h, in the presence or absence of 100 nM Liraglutide. Cells were harvested, and Bcl-2 phosphorylation [(**a**)] and BID expression [(**b**)] were determined by western blotting. *β*-Actin was used as the loading control to all western blot analyses. Each bar represents mean ± SEM from four independent experiments. Data is expressed as fold change to the control (CNTRL; unstressed/untreated conditions). Data was analysed by one- and two-way ANOVA, followed by *post hoc* Bonferroni’s multiple comparison t-test (^*^ *p* ≤ 0.05, ^**^ *p* ≤ 0.01 & ^***^ *p* ≤ 0.001 compared to CNTRL; ^#^ *p* ≤ 0.05 & ^##^ *p* ≤ 0.01 compared to the corresponding thapsigargin-treated cells).

**Figure 7:**
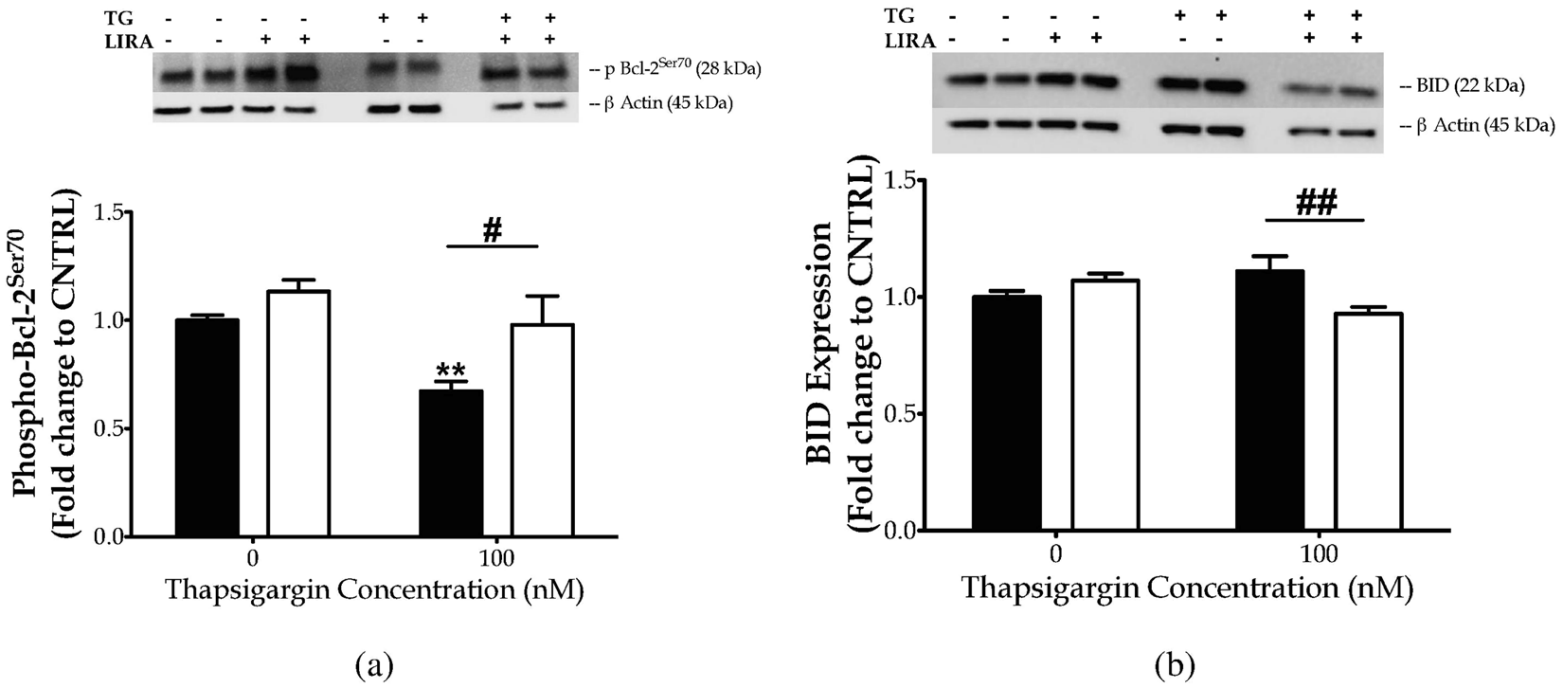
Chronic disturbance of ER calcium homeostasis diminishes Bcl-2 phosphorylation at serine 70 to promote cell apoptosis, though it does not affect the expression levels of the pro-apoptotic BID protein. Liraglutide restores Bcl-2 activation and further promotes a decrease in BID protein levels in the neuroblastoma SH-SY5Y cell line upon persistent ER stress. Twenty-four hours post seeding, SH-SY5Y cells were serum starved for 8 h and treated with 0 or 100 nM of thapsigargin for 16 h, in the presence or absence of 100 nM Liraglutide. Cells were harvested, and Bcl-2 phosphorylation [(**a**)] and BID expression [(**b**)] were determined by western blotting. b-Actin was used as the loading control to all western blot analyses. Each bar represents mean ± SEM from four independent experiments. Data is expressed as fold change to the control (CNTRL; unstressed/untreated conditions). Data was analysed by one- and two-way ANOVA, followed by post hoc Bonferroni’s multiple comparison t-test (^**^ *p* ≤ 0.01 compared to CNTRL; ^#^ *p* ≤ 0.05 & ^##^ *p* ≤ 0.01 compared to the corresponding thapsigargin-treated cells).

Our findings motivated us to scan for the signalling network that underlies the cross-talk among the different sub-cellular compartments and mediates the neuroprotective/restorative effects of Liraglutide [Figs 6 and 7, Table S1]. Sandwich-based antibody array has revealed that persistent disturbance of ER calcium homeostasis significantly precludes the activating phosphorylation of the extracellular signal-regulated kinase 1/2 (ERK1/2) at threonine 202 (Thr202) and tyrosine 204 (Tyr204) residues (*p* ≤ 0.05) and of Stat3 at Tyr705 (*p* ≤ 0.01), as shown in Fig. 6(b),(f), respectively. It additionally induces the glycogen synthase kinase 3*β* (GSK3*β*) [Fig. 6(d)] by halving the inhibitory phosphorylation of the kinase at the serine 9 (Ser9) residue (*p* ≤ 0.001), and significantly impedes the phosphorylation of the stress-responsive p53 at Ser15 (*p* ≤ 0.05) [Fig. 6(e)]. All these alterations in the signalling molecules further correlate to a two-fold increase (*p* ≤ 0.001) in cleaved poly (ADP-ribose) polymerase (PARP) [Fig. 6(h)] that assures the fulfilment and irreversibility of the apoptotic process^34^ upon a 16- time course of thapsigargin treatment in SH-SY5Y cells. Liraglutide rescues STAT3 activation (*p* ≤ 0.05) [Fig. 6(f)] but does not alleviate ERK1/2 impairments [Fig. 6(b)]. Moreover, Liraglutide restores GSK3*β* [Fig. 6(d)] and p53 [Fig. 6(e)] activity to the baseline levels, and significantly provokes Akt phosphorylation at Thr308 (*p* ≤ 0.05) under chronic thapsigargin treatment in the SH-SY5Y cells [Fig. 6(c)]. Liraglutide significantly attenuates ectopic PARP proteolysis (*p* ≤ 0.05) upon unmitigated UPR and autophagy impairments that signifies cytoprotection, though it remained significantly elevated when compared to control conditions (*p* ≤ 0.05) in *post-hoc* analysis [Fig. 6(h)].

It is well documented that the majority of pro-death signals emerging from UPR regulate the expression and activity of pro- and anti-apoptotic proteins of BCL-2 family^6,8^. Consistently, we report that persistent ER-localised blocking of calcium flux diminishes the inhibitory phosphorylation of the pro-apoptotic BAD at Ser112 (*p* ≤ 0.05) [Fig. 6(g)], accompanied by a significant de-phosphorylation of the survival-agonist Bcl-2 at Ser70 (*p* ≤ 0.01) [Fig. 7(a)]. Liraglutide normalises aberrant BAD [Fig. 6(g)] and Bcl-2 [Fig. 7(a)] phosphorylation patterns and further down-regulates the normal expression of the full-length BH3 interacting-domain death agonist (BID) [Fig. 7(b)].

## Discussion

Neuronal injury owing to chronic and irremediable ER stress has been increasingly correlated with a range of neurodegenerative disorders while UPR activation and deregulation has been repeatedly found in postmortem brain samples from affected patients and animals of AD, PD, ALS, HD, and experimental stroke^1–5^. Pharmacological and genetic manipulation approaches have unravelled promising mechanisms that link ER stress to neurodegenerative processes. In particular, AD features the accumulation of amyloid-*β* (A *β*) peptides in the brain, which underlies neuronal dysfunction and cognitive decline. A*β* primarily perturbs the cellular redox status and abnormally increases the amount of calcium that can be released by the ER, triggering ER stress, mitochondrial dysfunction and thereby neuronal toxicity and astrogliosis *in vivo* ^1,4^. Accordingly, *α*-Synuclein, the molecular determinant of PD pathobiology, alters the interactions between ER and mitochondria, which triggers an aberrant increase in the mitochondrial calcium content and compromises mitochondrial membrane potential, autophagic function and cellular bioenergetics^35^. Recent studies have further revealed that *α*-synuclein preferentially accumulates within the ER\microsomes, where it aggregates in toxic oligomeric formations in mouse and human brain with *α*–synucleinopathy. Those oligomeric forms have been further associated with the onset of chronic ER stress and disease progression^36,37^. UPR and ER calcium dynamics seem to additionally govern neuronal injury progression in HD^38,39^, TBI and following stroke^3^. Therefore, resolution of chronic ER stress can benefit acute and chronic neurodegenerative disorders that feature diverse aetiologies and clinical manifestations^3–5^, whilst prolonged perturbation of ER calcium homeostasis may offer an integrated cellular model to simulate neurodegenerative processes for drug discovery.

GLP-1 analogues, which are currently approved for T2DM treatment, have been repeatedly shown to exert neurotrophic/restorative effects in a range of animal models of AD, PD, ALS, TBI and experimental stroke^11–18,40–48^. Importantly, GLP-1 mimetics, such as Liraglutide has rescued the AD-related reduction in cortical activity and energy utilisation^22^, and a recently phase II clinical trial testing Exenatide has impeded PD progression^25^. The underlying biochemical processes are manifold. Incretin mimetics have prevented aberrant apoptosis of (hippocampal and primary cortical and dopaminergic) neurons and SH-SY5Y neuroblastoma cells exposed to hypoxia, excitotoxic insults, neurotoxins (*e.g*., hydrogen peroxide and oxidopamine) and thapsigargin-induced ER stress^17,18,49,50^. Liraglutide pre-treatment favours cell survival over apoptotic signalling to promote cytoprotection from persistent mitochondria dysfunction in SH-SY5Y cells^21^. Similarly, post-treatment with GLP-1R agonists rescues aberrant cytotoxicity and impaired viability, and further enhances cell survival signalling to protect SH-SY5Y neuroblastoma cells from chronic rotenone-induces oxidative stress^19^. In the present study, we have addressed the neuroprotective/restorative effects of Liraglutide, along with the underlying molecular mechanisms and signalling network after prolong perturbation of ER calcium homeostasis. Consistently, we report that Liraglutide impedes the increase in the number of SH-SY5Y cells with compromised plasma membrane and mitochondrial dysfunction induced by thapsigargin, and promotes cell proliferation in a stressor dose-dependent manner.

Thapsigargin is a specific, almost irreversible inhibitor of the SERCA channel, triggering a transient increase in the cytosolic calcium and depleting ER calcium stores^28^. Calcium depletion in the ER precludes the activity of calcium-dependent chaperones to potentiate the accumulation of unfolded/misfolded proteins within the organelle lumen and thereby ER stress^7^. In response, the cell activates the UPR network that integrates signals about the chronicity and severity of the stress stimuli and culminates into disproportionate activation of PERK, ATF6 and IRE1*α* signalling to determine cell fate^6,8,51^. Particularly, chronic ER stress augments PERK arm to amplify the transcription and translation of the pro-apoptotic transcription factor CHOP^51^, whilst it suppresses IRE1*α* signalling^51,52^ to possibly attenuate the survival and neurotrophic effects of the downstream spliced X-box binding protein 1(XBP-1) and sensitise the cells to ER stress^8^. It can additionally enhance ATF6 activity to reinforce CHOP expression and thus amplify the apoptotic component of the UPR^8,51^. Consistently, we have demonstrated that chronic thapsigargin treatment up-regulates ATF6 which accumulates in the cell nuclei and signifies its activation, whilst almost abolishes the activating phosphorylation of IRE1*α* at Ser724. We additionally show that chronic thapsigargin treatment triggers an ectopic expression of CHOP that correlates with calnexin deficiency and ERO1*α* excess in SH-SY5Y neuroblastoma cells. Calnexin silencing has been previously reported to sensitise cardiomyocytes to ER stress and favour apoptosis through CHOP up-regulation and calcium deregulation^53^. Furthermore, induction of the oxidoreductase ERO1*α* downstream of CHOP perturbs the ER redox state^54^ that in turn stimulates inositol-1,4,5-trisphosphate receptor (IP_3_R)-mediated calcium efflux into cytosol^55^. The latter could influence multiple pathways upstream of the core apoptosis machinery and, most importantly, mitochondrial function^56,57^. In addition to CHOP, persistent ER stress can activate the ER-resident CASP12 to further promote cell suicide. CASP12 provokes a downstream caspase cascade that leads to PARP degradation and therefore programmed cell death initiation^58–60^, as reflected in our results too. Intriguingly, Liraglutide co-treatment normalises BiP induction along with ATF6 and IRE1*α* signalling in thapsigargin-treated SH-SY5Y cells. It additionally mitigates abnormal CHOP expression and CASP12 activity, restores calnexin and ERO1*α* expression, and alleviates PARP degradation. These biochemical traits further relate to the restoration of SH-SY5Y cell viability and proliferation. Collectively, our findings suggest that GLP-1R activation resolves the induction of UPR effectors and pro-apoptotic mediators and promotes chaperone homeostasis in the ER lumen, which signifies cell proteostasis upon persistent, neuronal ER stress.

Acute SERCA channel inhibition has been shown to preclude autophagosome formation^30^ and fusion with lysosomes^31^, resulting into autophagy arrest^30,31^. Autophagy fail along with deregulated UPR seem to drive the imbalance between protein generation and degradation that underlies the onset and progression of neuronal degeneration^1,5,32^. Autophagy is a tightly regulated pathway that allows cells to eliminate harmful or damaged components through catabolism and recycling to maintain nutrient and energy homeostasis. As such, autophagy constitutes a crucial mechanism for preserving structures and functioning of subcellular organelles, including ER and mitochondria, when operates at basal levels, and for cell survival in response to stress^1,5,32^. Several studies have shown that Liraglutide promotes autophagy and thereby cell survival in liver, pancreas and SH-SY5Y cells^19,61–66^. Contrarily, Zhao *et al*. have demonstrated a cytoprotective effect of Liraglutide by inhibiting autophagy in renal tubular epithelial cells^67^. Similarly, Liraglutide has prevented oxidative stress-induced axonal injury by halting excessive autophagy in retinal ganglion cells^68^. Excessive autophagic flux can exert detrimental effects by aberrantly degrading endogenous inhibitors of apoptosis and Atg components, and lead to cell death. That dual role has been further attributed to autophagy under ER stress conditions^32^. Here, we show that apoptotic ER stress correlates with a substantial decrease in the protein levels of beclin-1, which determines the initiation and formation of phagophore. Suppressed IRE1*α* and Bcl-2 phosphorylation may underlie beclin-1 impairments following prolonged thapsigargin-treatment in SH-SY5Y cells. Previous studies have revealed that, among the three UPR arms, the induction of pro-survival autophagy after ER stress requires IRE1 signalling^69^. The latter mediates the phosphorylation of Bcl-2, which results in its dissociation from and to the release of beclin-1^70^. In addition, spliced XBP1, lying downstream of IRE1 arm, has been previously shown to bound to the promoter of beclin-1 and induce its transcription^71^. Furthermore, we demonstrate that chronic ER calcium dyshomeostasis culminates into Atg3, Atg7 and LC3 protein deficiency that may result from the ectopic CHOP expression. Indeed, CHOP has been previously shown to limit autophagy through the transcriptional control of a dozen of Atg genes involved in phagophore elongation and maturation into the autophagosome, and thereby to stimulate apoptosis upon persistent ER stress^72,73^. Autophagy dysfunction along with persistent ER stress can further trigger the excess accumulation of the autophagy adaptor protein p62^74^, which contains a KEAP1 binding motif similar to Nrf2^75^. Accumulation of p62 leads to KEAP1 sequestration and inactivation, which, in turn, promotes aberrant nuclear Nrf2 localisation and transcription of Nrf2 target genes^75^. Although the Nrf2 transcription program has been recognised as one of the main antioxidant defensive mechanisms for cytoprotection^33^, that constitutive Nrf2 activation along with autophagy preclusion has been previously shown to drive the hepatic injury observed in Atg7-knockout mice^75^. Accordingly, we report that the deficiency in the “core” Atg components, lying downstream of prolong ER calcium perturbation, correlates with Nrf2 hyperactivaiton, suggesting that regulated Nrf2 activation can benefit cellular defensive responses to stress. Strikingly, Liraglutide co-treatment ameliorates Atg3, Atg7, beclin-1, and LC3 expression impairments and further normalises aberrant Nrf2 and Bcl-2 activity in thapsigargin-treated SH-SY5Y cells. Taken together, our findings highlight that GLP-1R activation promotes ER and autophagic machinery homeostasis that signifies about cell adaptation and survival upon unmitigated, neuronal ER stress.

Mechanistically, Liraglutide restores impaired STAT3 signalling and engages Akt pathway to exert its neuroprotective/restorative effects upon persistent ER stress. STAT3 is a latent transcription factor that is primarily activated by the phosphorylation of a single tyrosine residue, Tyr705, in response to the stimulation of cytokine and growth factor receptors^76,77^, including GLP-1R^78^. Once activated, STAT3 dimerises and enters the nucleus to orchestrate – alone or in cooperation with other factors – transcriptional programs for neuronal/glial survival, proliferation and differentiation^76^. Suppressed STAT3 activity has been previously found in the hippocampus of postmortem brain samples from AD patients and transgenic mice^79^. Chiba *et al*. have further demonstrated that A*β* inactivates hippocampal JAK2 (Janus kinase 2)/STAT3 axis to provoke the basal forebrain cholinergic dysfunction and spatial working memory deficits *in vivo*, linking STAT3 pathway to neurodegenerative processes^79^. It has been additionally recognised that ER stress perturbs JAK-signal transducers and STAT3 signalling to mediate acute-phase neuroinflammatory responses^80^ and leptin resistance in the brain^81^ or to drive apoptosis that underlies suppressed hepatic gluconeogenesis^82^ in periphery. Although the mechanistic interplay among UPR, STAT3 signalling and GLP-1R in the context of cell fate and neuronal degeneration remains unknown, STAT3 has been recently emerged as a multifaceted determinant for autophagy^77^. For instance, nuclear STAT3 can transcriptionally induce the anti-apoptotic Bcl-2 expression and consequently inhibit autophagy^83^. However, this phenotype is not prominent on our findings as no changes in total Bcl-2 levels occur following chronic thapsigargin and/or Liraglutide treatments in SH-SY5Y cells [Figure S1]. Intriguingly, Atg3 promoter has been recently shown to contain a STAT3-responsive element^84^, which may explain the restorative effects of Liraglutide against the thapsigargin-induced Atg3 deficiency, though this hypothesis requires further investigation.

Akt (also known as protein kinase B – PKB) is a serine/threonine kinase member of the AGC protein kinase family with a profound function in growth, proliferation, intermediate metabolism and cell survival^85^, and a pivotal effector of the anti-apoptotic GLP-1R signalling^21^. In response to the phosphoinositide 3-kinase (PI3K) stimulation, Akt is recruited to the cell membrane^86^ where it can undergo phosphorylation at the threonine 308 (Thr308) residue by PDK1^87^ and at the serine 473 (Ser473) residue by mTORC2^88^. Phosphorylation of Thr308 site critically determines Akt activation whilst phosphorylation of both aforementioned sites is required for the maximal kinase activity^86,89^. In our study, Liraglutide has significantly increased the phosphorylating levels of Akt at Thr308 that signifies kinase activation. Activated Akt phosphorylates multiple targets in the cytoplasm, nucleus, mitochondria and ER membrane to regulate adaptive responses and cell fate under diverse insults, including ER and oxidative stress and DNA damage^85,89–91^. Among others, Akt phosphorylates and inhibits the death-agonist BAD that becomes rapidly de-phosphorylated upon apoptotic stimuli^89^, as prominent in our findings too. Active (de-phosphorylated) BAD binds to the survival-agonist Bcl-x_*L*_ or Bcl-2 at the mitochondria that provokes Bax and Bak oligomerisation and perturbs mitochondrial membrane permeabilisation to favour the point-of-no-return of apoptotic cell death^92–94^. However, the restoration of BAD phosphorylation downstream of growth factor signalling raises the mitochondrial threshold for apoptosis that renders the cells less vulnerable to death signals^95^, as evident in our results too.

In addition to BAD, Akt phosphorylates and inhibits the GSK3*β*, a major protein kinase that drives neurodegenerative processes in AD^96^ and neuronal apoptosis following ER stress^97–100^. Indeed, accumulating evidence from diverse neuronal cell lines, primary neuronal cultures, and ER insults has demonstrated that the UPR abolishes the inhibitory phosphorylation of GSK3*β* at Ser9^97–100^ to promote CHOP expression and switch from pro-survival to pro-death signalling during ER stress^98^, as reflected in our results too. Moreover, it has been previously reported that PERK engages GSK3*β* to phosphorylate the p53 tumour suppressor protein at Ser315 and Ser376 and favour nuclear export and proteosomal degradation of p53 upon ER stress^101,102^. p53 is transcription factor of which activation serves to organise cellular responses with apoptosis, cell cycle arrest, senescence, DNA repair, cell metabolism, or autophagy depending on the nature and degree of stress insult, environmental context, and cell type. The regulation of p53 is complex and involves post-translational modifications – *e.g*., phosphorylation and acetylation – at multiple sites that impact its cellular localisation, stability and transcriptional activity^103^. We assessed the phosphorylated levels of p53 at Ser15 that facilitates p53 nuclear accumulation and stabilisation by halting the ability of the E3 ubiquitin-protein ligase Mdm2 to interact with and target p53 for proteosomal degradation^104,105^. Our findings indicate that chronic ER stress precludes the phosphorylation of p53 at Ser15 and further confirm that ER stress leads to p53 destabilisation^101,102^, though different phosphorylation sites were examined among the studies. In our study, the p53 destabilisation seems to lie downstream of the decreased activity of ERK1/2, which has been previously shown to regulate the phosphorylation of this transcription factor at Ser15 in SH-SY5Y cells^106^. Although Liraglutide does not alleviate the impaired ERK1/2 phosphorylation, it potentiates Akt signalling which has been previously shown to potentiate the atypical p53-related protein kinase and phosphorylate p53 at Ser15 in human cell lines^107^, as well as to restore p53 stabilisation following cellular stress^108^. Intriguingly, it has been recently reported that Akt phosphorylates and inhibits PERK^109^, which may offer an additional mechanistic link on how Liraglutide confers its restorative effects on p53, though necessitates further experimentation.

Though not examined in the present study, Liraglutide may restore calcium homeostasis to elicit neuroprotection upon chronic thapsigargin treatment. Previous studies have demonstrated that GLP-1R induction potentiates cyclic AMP (cAMP) production^20^ and regulates calcium responses^21,49^, which underlie protection of hippocampal neurons and SH-SY5Y neuroblastoma cells from excitotoxicity^49^ and oxidative stress-induced^21^ apoptosis, respectively. Furthermore, cAMP increase by GLP-1R stimulation potentiates protein kinase A (PKA) that downstream induces SERCA function to promote cytoprotection in insulin-resistant macrophages^110^ and high glucose-treated cardiomyocytes^111^. Accordingly, PKA pharmacological inhibition blocks the GLP-1R-mediated anti-apoptotic effects, whilst the adenylate cyclase activator, Forskolin mimics the GLP-1R-induced cardioprotection upon hyperglycaemia^111^. cAMP has been additionally shown to potentiate cAMP-regulated guanine nucleotide exchange factors (also known as Epac) that regulate calcium dynamics in response to GLP-1R stimulation^112^.

In conclusion, our study demonstrates the neuroprotective/restorative effects of Liraglutide upon unmitigated neuronal ER stress. It further unravels a complex signalling network through which Liraglutide regulates UPR outcome, elicits autophagy machinery homeostasis and shifts cell fate from apoptosis to survival, providing additional evidence for the beneficial effects of GLP-1R stimulation and signalling in neurodegenerative disorders and deepening our understanding of the underlying mechanism.

## Materials and Methods

### Materials

Cell proliferation kit II [2,3-Bis-(2-methoxy-4-nitro-5-sulphophenyl)-2H-tetrazolium-5-carboxanilide (XTT)], cell proliferation ELISA 5-bromo-2′-deoxyuridine (BrdU) kit and Cytotoxicity Detection Kit^PLUS^ [lactate dehydrogenase (LDH)] were purchased from Roche Diagnostics Ltd (West Sussex, UK). The ER Stress Antibody Sampler Kit (#9956), beclin-1 (D40C5) monoclonal antibody (#3495), Atg3 monoclonal antibody (#3415), Atg7 monoclonal antibody (#8558), LC3B polyclonal antibody (#2775), Bcl-2 (D17C4) monoclonal antibody (#3498), phospho-Bcl-2 (Ser70) (5H2) monoclonal antibody (#2827), BID (Human Specific) polyclonal antibody (#2002), *β*-Actin (8H10D10) monoclonal antibody (#3700), PathScan^®^ Intracellular Signaling Array Kit (#7323), PathScan^®^ Sandwich ELISA Lysis Buffer (1X), Protease/Phosphatase Inhibitor Cocktail (100X), and horseradish peroxidase(HRP)-linked secondary antibodies against the corresponding species IgG of the primary antibodies (#7074 & #7076) were purchased from Cell Signaling Technology (New England Biolabs UK Ltd, Hertfordshire, UK). Phospho-IRE1 (S724) polyclonal antibody (ab48187), ATF6 [1-7] polyclonal antibody (ab122897), CASP12 polyclonal antibody (ab62484), Nrf2 [EP1808Y] (ab62352) monoclonal antibody and normal goat serum were purchased from Abcam (Cambridgeshire, UK). Quick Start^™^. Bradford protein assay kit was obtained from BIO-RAD Laboratories Ltd (Hertfordshire, UK). Amersham ECL Prime western blotting detection reagent kit was obtained from GE Healthcare Life Sciences (Buckinghamshire, UK). Invitrogen^®^ goat anti-rabbit IgG H & L Alexa Fluor^®^ 488 secondary antibody (A11034), Invitrogen^™^ iBlot^®^ 2 Dry Blotting System, iBlot^™^ 2 Transfer Stacks with integrated nitrocellulose transfer membranes, precast polyacrylamide Bolt^™^ 4–12% gradient Bis-Tris Plus gels, and Restore^™^ PLUS Western Blot Stripping Buffer were purchased from Fisher Scientific UK Ltd (Leicestershire, UK). Bovine serum albumin (BSA), tris buffered saline (TBS; pH 8.0) supplemented or not with 0.05% Tween^®^ 20, phosphate buffered saline (PBS; pH 7.4), paraformaldehyde and dimethyl sulfoxide (DMSO; anhydrous, ≥ 99.9%) were obtained from Sigma-Aldrich Company Ltd (Dorset, UK). Other materials and reagents for cell culture, western blotting and immunocytochemistry were purchased from Fisher Scientific UK Ltd (Leicestershire, UK), unless otherwise stated. No ethical approval was required for the current work.

### Cell culture

The human neuroblastoma SH-SY5Y cell line (ATCC^®^ CRL2266^™^) was obtained from LGC Standards (Middlesex, UK) and cultivated in Dulbecco’s modified eagle medium/nutrient mixture F-12 (DMEM/F-12, 1:1; 1X) Glutamax^™^ supplemented with 10% heat-inactivated foetal bovine serum (FBS), 100 IU ml^−1^ of Penicillin and 100 μg ml^−1^ of Streptomycin. Cells were maintained at 37 °C in a humidified incubator with 5% CO_2_ and 95% air. Cells were subcultured when 80–90% confluent and seeded at 1:10 ratio. When passaged, viable cells were counted and seeded at the desired cell density for the assays using the Countess^™^ Automated Cell Counter (Thermo Fisher Scientific, Inchinnan Business Park, Paisley, UK). The latter is based on the standard trypan blue exclusion technique, in which dead cells are selectively permeable to the dye and stained blue. Culture medium was renewed every 3 to 4 days.

### Cell treatments

Thapsigargin was received as a colourless solid film, solubilised in 100% dimethyl sulfoxide (DMSO) at a concentration of 1 mM, aliquoted and stored at  −20 °C until used. For the experiments, thapsigargin stock preparations were serially diluted in serum-free culture medium at final working concentrations of 10 to 1000 nM, containing ≤0.1% DMSO; DMSO (≤0.1%) did not affect cell viability and proliferation as assessed in preliminary experiments (data not shown).

Liraglutide was purchased from GL Biochem Ltd (Shanghai, China). The purity of the peptide was analysed by reverse-phase HPLC and characterised using matrix-assisted laser desorption ionisation time-of-flight (MALDI - TOF) mass spectrometry, as previously described^113^. The peptide was reconstituted in Gibco Water for Injection for Cell Culture to a concentration of 1, aliquoted and stored at −20 °C until used. For the experiments, Liraglutide stock preparations were diluted in serum-free culture medium to a final working concentration of 100 nM. The concentration was selected on the basis of previous experiments in which our group has established optimal working concentrations for the neuroprotective and anti-apoptotic effects of Liraglutide^19,21^.

### Cell viability, proliferation and cytotoxicity assessment

Cell viability, proliferation and cytotoxicity were determined using Cell proliferation kit II (XTT), cell proliferation ELISA BrdU (colorimetric) kit and Cytotoxicity Detection Kit^PLUS^ (LDH), respectively. The assays were formatted in Nunc^™^ MicroWell^™^ flat-bottomed 96-well plates. SH-SY5Y cells were seeded at a density of 2 × 10^4^ cells per well for 24 h. The cells were subsequently serum starved in serum-free medium for 8 h and stressed with different concentrations of thapsigargin for 16 h, in the presence or absence of 100 nM of Liraglutide. All cell treatments were performed in sextuplicate per plate per experiment. Following cell treatments, 50 μL of XTT labelling reagent and 1 μL of electron coupling reagent was added to each well, yielding final XTT concentration of 0.3 mg mL^−1^. Cells were incubated with the XTT labelling mixture for 6 h at 37 °C in a humidified incubator with 5% CO_2_ and 95% air. Plates were then gently shaken for 5 min on a Microtitre plate shaker (Stuart, Staffordshire, UK) and absorbance was measured at 492 and 690 nm (reference wavelength) in a Infinite^®^ 200 PRO microplate reader (Tecan, Berkshire, UK). The assay rests on the cleavage of the yellow tetrazolium salt XTT into an orange, water-soluble formazan product by metabolically active cells (mitochondrial dehydrogenase enzyme activity). Thus, absorbance values are proportional to the number of viable cells in the respective microcultures.

Alternatively, SH-SY5Y cells were incubated with 10 μM BrdU labelling solution for 6 h at 37 °C in a humidified incubator with 5% CO_2_ and 95% air. The pyridine analogue BrdU was incorporated, in place of thymidine, into the newly synthesised DNA strands of proliferating cells. BrdU incorporation was detected by immunoperoxidase staining and a subsequent colorimetric substrate reaction as per manufacturer’s protocol. Plates were read at 450 and 690 nm (reference wavelength) using Infinite^®^ 200 PRO microplate reader. Developed colour and absorbance values reflect the amount of DNA synthesis, and hereby the number of proliferating cells in the respective microcultures.

Cytotoxicity Detection Kit^PLUS^ was used to quantify LDH activity; LDH is a stable cytoplasmic enzyme present in all cells and rapidly released into the culture supernatant upon plasma membrane damage. The assay involves a coupled enzymatic reaction that results in the formation of a red, water-soluble formazan product during a limited time period. The amount of colour developed is proportional to the number of damaged/lysed cells in the respective microcultures. As per manufacturer’s instructions, following cell treatments, 50 μL of cell supernatant was collected and incubated with the provided reaction mixture for 30 min at room temperature. The reaction was terminated by adding the Stop Solution from the kit and absorbance was measured at 490 and 650 nm (reference wavelength).

### Sample collection and protein extraction

2×10^6^ cells were grown at 75 cm^2^ Nunc^™^ Cell Culture Treated EasYFlasks^™^ for 24 h. Following serum starvation for 8 h, cells were stressed with 100 nM of thapsigargin for 16 h, in the presence or absence of 100 nM of Liraglutide. Thereafter, cells were washed once with ice-cold 1X phosphate-buffered saline (PBS) and harvested in 1X cell lysis buffer containing protease/phosphatase inhibitor cocktail (1X). After two freeze/thaw cycles, whole-cell lysate was collected and total protein was extracted by centrifugation at ~16000×*g* at 4 °C for 15 min. Quick start^™^ Bradford protein assay was conducted to estimate the protein concentration of the samples, as previously described^19,21^.

### Western blotting

Protein of whole-cell lysate (4 μg) was reduced and denaturated by boiling in lithium dodecyl sulfate (LDS) sample buffer containing 50 mM dithiothreitol (DTT) at 95 °C for 5 min. Replicate protein samples were separated on Bolt^™^ 4–12% gradient Bis-Tris gel and blotted onto nitrocellulose membranes using iBlot^®^ 2 Dry Blotting System. Blots were washed once in 1X TBS for 5 min, blocked in 5% w/v skimmed milk for 1 h at room temperature, and probed with the primary antibodies against BiP, calnexin, Ero1*α*, PDI, IRE1*α*, CHOP, ATF6 (1:500), CASP12 (1:2000), BID, LC3, ATG3, ATG7, beclin, and *β*-actin (1:10^4^) overnight at 4 °C. Alternatively, blots were blocked in 5% BSA and probed with the primary antibodies against phospho-IRE1 (Ser724) and phospho-Bcl-2 (Ser70) overnight at 4 °C. All the primary antibodies used were diluted in 5% BSA in 1X TBS with 0.05% Tween^®^ 20 (TBS–T; pH 8) at 1:1000 ratio, unless otherwise specified. All the primary antibodies used were generated in rabbit, except for the antibodies against CHOP, ATF6 and *β*-actin which were raised in mice. Following primary antibody incubation, blots were washed three times in 1X TBS–T for 5 min each and incubated with the HRP-linked secondary antibodies against the corresponding species IgG (1:2000) for 1 h at room temperature. Blots were developed using Amersham ECL Prime western blotting detection reagent kit as per manufacturer’s instructions. ChemiDoc^™^ MP Imaging System with Image Lab^™^ software (BIO-RAD Laboratories Ltd, Hertfordshire, UK) used to image chemiluminescent bands and perform densitometric analysis. *β*-Actin protein was served as loading control to which relative peak intensities of the examined markers were normalised. To reprobe, blots were incubated in Restore^™^ PLUS stripping buffer for 20 min at 37 °C with gentle agitation and subsequently washed three times in TBS for 5 min each. Chemiluminescent detection to ensure the removal of the original signal preceded blot re-incubation with another primary antibody of interest.

### PathScan^®^ sandwich immunoassay

The PathScan^®^ Intracellular Signaling Array kit was used in accordance to manufacturer’s protocol. Briefly, 100 μL Array Blocking Buffer was added to each well of the the antibody array slide and incubated for 15 min at room temperature. Thereafter, protein of whole-cell lysate (0.3 mg mL^−1^) was placed onto each well and incubated overnight at 4 °C with gentle agitation. All samples per experiment were processed in duplicate. The following day, all the wells were washed three times in 1X Array Wash Buffer for 5 min each, and probed with 1X Detection Antibody Cocktail for 1 h at room temperature with gentle agitation. Streptavidin-conjugated HRP along with LumiGLO^®^/Peroxide Reagent were used to visualise the bound antibody cocktail by chemiluminescence. ChemiDoc^™^ MP Imaging System with Image Lab^™^ software was used to capture images of the slide. The Protein Array Analyzer^114^ for Image J (National Institutes of Health, Bethesda, Maryland, USA) was used for the densitometric analysis. The relative peak intensities of the examined signalling molecules were normalised to the corresponding values of the positive and negative controls of the array.

### Immunocytochemistry

1×10^5^ cells per well were grown on the Millicell EZ SLIDE eight-well glass chamber slides for 24 h. Following serum starvation for 8 h, cells were stressed with 100 nM of thapsigargin for 16 h, in the presence or absence of 100nM of Liraglutide. Thereafter, cells were washed once with 1X PBS, fixed with 4% paraformaldehyde for 10 min and permeabilised in 0.3% Triton-X-100 for 5 min at room temperature. Cells were blocked in 10% normal goat serum and incubated with the primary antibody against Nrf2 (1:500) or ATF6 (1:200) overnight at 4 C. Primary antibody was diluted in 1% BSA in 1X PBS supplemented with 0.3% Triton-X-100. Following primary antibody incubation, cells were washed three times in 1X PBS for 5 min each and incubated with goat anti-rabbit IgG H & L Alexa Fluor^®^ 488 secondary antibody (1:1000) for 1 h at room temperature. Specimens were cover-slipped with Vectashield Antifade Mounting Medium with DAPI (Vector Laboratories Ltd, Cambridgeshire, UK) and sealed with nail polish. Zeiss LSM510 Meta Laser Scanning confocal microscope was used for cell imaging and eight pictures were captured per experimental group per experiment for quantification. Image J was used to quantify Nrf2 corrected total cell fluorescence (CTCF) in the cell nucleus. Image acquisition and processing were performed in a blinded fashion.

### Statistics

All the results were expressed as mean ± standard error (SEM) of at least three independent experiments for each group. Differences among means were considered significant if *p* ≤ 0.05. Data was processed with one way ANOVA analysis, followed by *post hoc* Bonferroni’s multiple-comparison t-tests to identify differences among groups of unstressed and stressed conditions. The effects of Liraglutide were studied by two way ANOVA, followed by *post hoc* Bonferroni’s multiple-comparison t-tests. Statistical calculations were performed in GraphPad Prism 5 (GraphPad Software Inc., San Diego, USA) for Mac OS X software.

### Data availability

The data supporting the findings of this study are included in this published article and its supplementary information file. All datasets generated during the current study are available from the corresponding author (C.H.) on reasonable request.

## Electronic supplementary material

Supplementary Information
