# Supplementary material for: Liraglutide restores chronic ER stress, autophagy impairments and apoptotic signalling in SH-SY5Y cells
Source: Sci Rep. 2017 Nov 23;7:16158. doi: 10.1038/s41598-017-16488-x (PMC5700973; doi:10.1038/s41598-017-16488-x)
Supplement: Supplementary file 1 — Supplementary Information [file 41598_2017_16488_MOESM1_ESM.pdf]

Liraglutide restores chronic ER stress, autophagy impairments and apoptotic signalling in SH-SY5Y cells

Theodora Panagaki[1], Maria Michael[1], and Christian Hölscher[1,*]

[1]Biomedical & Life Sciences Division, Lancaster University, Lancaster, LA1 4YG, UK
[*]c.holscher@lancaster.ac.uk

Supplementary Information

**Target**	**Modification Site**	**Mean ± SEM**				**One-way ANOVA**	
		CNTRL	LIRA	TG	TG + LIRA	F	p
Stat1	Tyr 701	1.00 ± 0.178	1.36 ± 0.106	1.02 ± 0.162	1.38 ± 0.194	$(3, 28) = 1.619$	0.2072
Akt	Ser 4731	1.00 ± 0.214	1.42 ± 0.241	0.867 ± 0.151	1.23 ± 0.160	$(3, 28) = 1.575$	0.2174
AMPKα	Thr 172	1.00 ± 0.201	0.962 ± 0.191	0.666 ± 0.056	0.619 ± 0.052	$(3, 28) = 1.870$	0.1575
S6 Ribosomal Protein Kinase	Ser 235/ 236	1.00 ± 0.1664	0.813 ± 0.095	0.733 ± 0.096	0.958 ± 0.076	$(3, 28) = 1.203$	0.3268
mTOR	Ser 2448	1.00 ± 0.107	1.02 ± 0.122	0.70 ± 0.103	1.15 ± 0.190	$(3, 27) = 2.018$	0.1351
HSP27	Ser 78	1.00 ± 0.241	0.970 ± 0.145	0.609 ± 0.082	0.635 ± 0.128	$(3, 28) = 1.727$	0.1841
p70 S6K	Thr 389	1.00 ± 0.103	0.863 ± 0.062	0.930 ± 0.122	0.807 ± 0.115	$(3, 28) = 0.6552$	0.5864
PRAS40	Thr 246	1.00 ± 0.176	0.925 ± 0.138	0.712 ± 0.077	0.798 ± 0.102	$(3, 28) = 0.9936$	0.4102
p38	Thr 180/Tyr 182	1.00 ± 0.212	1.03 ± 0.227	0.662 ± 0.140	1.16 ± 0.165	$(3, 28) = 1.261$	0.3068
SAPK/JNK	Thr 183/Tyr 185	1.00 ± 0.183	1.03 ± 0.164	0.89 ± 0.078	0.87 ± 0.111	$(3, 28) = 0.3143$	0.8149
**Caspase 3**	Asp 175	1.00 ± 0.0782	0.98 ± 0.119	1.35 ± 0.149	1.06 ± 0.047	$(3, 28) = 2.561$	**0.0750**

Table S1: **Protein expression of additional signalling targets included in the PathScan® Intracellular Signaling Array kit**. Twenty-four hours post seeding, SH-SY5Y cells were serum starved for 8 h and treated with 0 or 100 nM of thapsigargin (TG) for 16 h, in the presence or absence of 100 nM Liraglutide (LIRA). Cells were harvested, and 0.3 mg mL^{-1} protein of whole-cell lysate was processed with the PathScan® sandwich immunoassay. All samples per experiment were processed in duplicate. Data is expressed as fold change to the control (CNTRL; unstressed/untreated conditions) from four independent experiments.

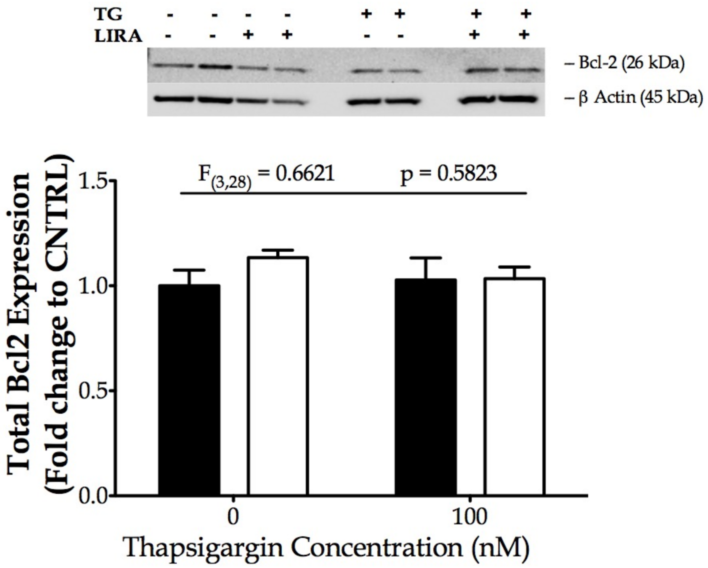

Figure S1: **Chronic disturbance of ER calcium homeostasis does not affect the expression levels of the pro-survival Bcl-2 protein.** Twenty-four hours post seeding, SH-SY5Y cells were serum starved for 8 h and treated with 0 or 100 nM of thapsigargin for 16 h, in the presence or absence of 100 nM Liraglutide. Cells were harvested, and Bcl-2 expression was determined by western blotting. β-Actin was used as the loading control to the western blot analysis. Each bar represents mean $\pm$ SEM from four independent experiments. Data is expressed as fold change to the control (CNTRL; unstressed/untreated conditions). Data was analysed by one- and two-way ANOVA, followed by *post hoc* Bonferroni's multiple comparison t-test.
